# Excessive daytime sleepiness is associated with an exacerbation of migraine: A population-based study

**DOI:** 10.1186/s10194-016-0655-4

**Published:** 2016-07-01

**Authors:** Jiyoung Kim, Soo-Jin Cho, Won-Joo Kim, Kwang Ik Yang, Chang-Ho Yun, Min Kyung Chu

**Affiliations:** Department of Neurology, Bio Medical Research Institute, Pusan National University Hospital, Pusan National University School of Medicine, Busan, South Korea; Department of Neurology, Dongtan Sacred Heart Hospital, Hallym University College of Medicine, Hwaseong, South Korea; Department of Neurology, Gangnam Severance Hospital, Yonsei University College of Medicine, Seoul, South Korea; Sleep Disorders Center, Department of Neurology, Soonchunhyang University College of Medicine, Cheonan Hospital, Cheonan, South Korea; Clinical Neuroscience Center, Department of Neurology, Seoul National University Bundang Hospital, Seongnam, South Korea; Department of Neurology, Kangnam Sacred Heart Hospital, Hallym University College of Medicine, 1 Singil-ro, Yeongdeungpo-gu, Seoul, 07441 South Korea

**Keywords:** Epidemiology, Excessive daytime sleepiness, Headache, Migraine, Sleep, Sleepiness

## Abstract

**Background:**

Previous studies have shown that migraine and sleep disturbances are closely associated. Excessive daytime sleepiness (EDS) is a common symptom of various types of sleep disturbance. Findings from clinic-based studies suggest that a high percentage of migraineurs experience EDS. However, the prevalence and clinical impact of EDS among migraineurs at the population level have rarely been reported. The objective of this study was to investigate the prevalence and impact of EDS among migraineurs using a population-based sample in Korea.

**Methods:**

We selected a stratified random sample of Koreans aged 19 to 69 years and evaluated them using a semi-structured interview designed to identify EDS, headache type, and the clinical characteristics of migraine. If the score on the Epworth Sleepiness Scale (ESS) was more than or equal to 11, the participant was classified as having EDS.

**Results:**

Of the 2,695 participants that completed the interview, 143 (5.3 %) and 313 (11.6 %) were classified as having migraine and EDS, respectively. The prevalence of EDS was significantly higher in participants with migraine (19.6 %) and non-migraine headache (13.4 %) compared to non-headache controls (9.4 %). Migraineurs with EDS had higher scores on the Visual Analogue Scale (VAS) for headache intensity (6.9 ± 1.8 vs. 6.0 ± 1.9, *p* = 0.014) and Headache Impact Test-6 (59.8 ± 10.2 vs. 52.5 ± 8.2, *p* < 0.001) compared to migraineurs without EDS.

**Conclusions:**

Approximately 20 % of migraineurs had EDS in this population-based sample. Excessive daytime sleepiness was associated with an exacerbation of some migraine symptoms.

## Background

Migraine and sleep disturbances are common neurological problems in the general population [1–3]. The close association between the two conditions has been reported in numerous clinic- and population-based studies. Both excessive sleep and sleep deprivation are common triggering factors of migraine and sleep may terminate an attack of migraine [4, 5]. Insomnia is a common comorbidity of migraine in cross-sectional and longitudinal studies [6, 7]. Habitual snoring and sleep bruxism are associated with an exacerbation of migraine [8, 9]. Morning headache in obstructive sleep apnea (OSA) patients may exhibit migraine-like headache [10]. Restless legs syndrome has been reported to be associated with migraine in case-controlled and population-based studies [11, 12].

Excessive daytime sleepiness (EDS) is defined as ‘sleepiness in a situation when an individual would be expected to be awake and alert’ [13]. EDS is not a disorder in and of itself, but represents symptoms of several broad conditions including poor sleep quality, depression, anxiety, sleep-disordered breathing, and obesity metabolic syndrome [14]. Besides impairing quality of life, EDS may lead to potentially problematic conditions such as attention deficits and sleep attacks [15]. The prevalence of EDS has ranged between 10 % and 20 % in the general population [16, 17].

For psychiatric conditions, anxiety and depression has been reported to be associated with EDS. Individuals with depression have frequently EDS (57.1 %) and a quarter of individuals with EDS have moderate-to-severe depression in survey using Beck Depression Inventory [18, 19]. Anxiety was associated with an increased occurrence of EDS in a 20-year prospective community-based study from Switzerland [17].

The association between EDS and several common neurological diseases such as Parkinson’s disease, epilepsy, and migraine has been previously reported. A case-controlled study from Italy noted that subjects with episodic migraine (EM) showed an increased odds ratio [14 % vs. 5 %; odds ratio (OR) = 3.1; 95 % confidence interval (CI) 1.1–8.9] for EDS compared to age- and sex-matched healthy controls [15]. Another case-controlled study showed a positive association between EDS and chronic migraine (CM) [20]. A case-series study in the United States showed that a significant proportion of participants with migraine also had EDS [21]. A community-based study in Norway showed increased ORs (OR = 3.3, 95 % CI 1.0–10.2) for EDS among migraineurs compared to headache-free individuals [22]. However, previous reports regarding the association between EDS and migraine were predominantly clinic-based studies and the association between them has seldom been examined in the general population. In addition, the clinical characteristics of migraine associated with EDS have not yet been reported.

The Korean Headache-Sleep Study (KHSS) is a nation-wide population-based interview survey regarding headache and sleep disturbance [12]. It provides an opportunity to assess comorbidity between EDS and migraine as well as elucidate the clinical characteristics of migraine relative to the presence of comorbid EDS. The present study investigated 1) the prevalence of migraine and/or EDS in a general population-based sample; 2) the comorbidity between migraine and EDS in association with potential covariates such as anxiety, depression and poor sleep quality; and 3) the clinical characteristics of migraine comorbid with EDS using the data available from the KHSS.

## Methods

### Study population and survey process

The study was a nationwide, cross-sectional survey of headache and sleep in the Korean population in adults aged 19–69 years. The study design and methods have been previously described in detail [12]. Briefly, we adopted a two-stage systematic random sampling method in all Korean territories except Jeju-do, proportional to the distribution of the population, which resulted in a sample of 2,695 individuals. Subjects were stratified according to age, gender, size of residential area and educational level. To minimize bias, we informed participants that the topic of the survey was social health issues rather than headache. Trained interviewers conducted structured interviews using a questionnaire to diagnose headache type, sleep duration, sleepiness, anxiety, and depression door-to-door using a face-to-face interview. The interview included questions on headache symptoms and sleep status. All interviewers were employed by Gallup Korea and had previous social survey interviewing experience. The study was conducted from November 2011 to January 2012. It was approved by the institutional review board/ethics committee of Hallym University Sacred Heart Hospital and was performed in accordance with the ethical standards described in the 1964 Declaration of Helsinki and its subsequent amendments [23]. Written informed consent was obtained from all participants.

### Migraine assessment

We diagnosed migraine using a questionnaire. The questionnaire established a headache profile which was designed to align with the second edition of the International Classification of Headache Disorders (ICHD-2) [24]. We investigated the severity of headache based on its effects on daily activity (mild, moderate, or severe) and using a visual analogue scale (VAS). Furthermore, we surveyed the headache frequency as following question. “How many days do you experience the headache every month?” Migraine was diagnosed based on the ICHD-2 criteria for migraine without aura (code 1.1) [24]. We did not attempt to separately diagnose migraine with and without aura. As such, both were included in the present study. The questions used to diagnose migraine have 75.0 % sensitivity and 88.2 % specificity, verified by comparing the diagnoses from the survey with those of doctors obtained from an additional telephone interview. This validation process has been previously described in detail [25].

### Non-migraine headache assessment

If a participant responded positively to the question ‘In the past year, have you had at least 1 headache lasting more than 1 min?’ and was not diagnosed as having migraine, she or he was diagnosed as having non-migraine headaches.

### Excessive daytime sleepiness, sleep duration and poor sleep quality assessment

We used the Epworth Sleepiness Scale (ESS) for assessing participants’ daytime sleepiness. The total ESS score ranged from 0 to 24. If a participant scored more than or equal to 11 on the ESS, they were classified as having EDS. The ESS has been previously validated in the Korean language [26]. The Korean version of the ESS has been reported to have good internal consistency (Cronbach’s α = 0.90) and test-retest reliability (*r* = 0.78 to 0.93)

We assessed the weekday and weekend sleep durations of participants. Average sleep duration was defined as [(weekday sleep duration × 5) + (weekend sleep duration × 2)]/7. Short sleep duration was defined as average sleep duration of less than 6 h and poor sleep quality was defined as a Pittsburgh Sleep Quality Index score (PSQI) of more than 5.

### Anxiety and depression assessment

We used the Goldberg Anxiety Scale (GAS) for measuring self-reported anxiety symptomology. If a participant provides a positive response for two or more of the first four screening questions and five or more of all GAS questions, they were characterized as having anxiety [27]. The Korean version of the GAS has been reported to have 82.0 % sensitivity and 94.4 % specificity [28].

The Patient Health Questionnaire-9 (PHQ-9) was used for diagnosing depression [29]. If a participant’s PHQ-9 score was 10 or more, they were categorized as having depression. The Korean PHQ-9 has been found to have 81.1 % sensitivity and 89.9 % specificity [30].

### Statistical analysis

We compared EDS prevalence among non-headache controls, and participants with non-migraine headache and migraine, using the chi-square test. We conducted a multivariable logistic regression analysis, adjusting for anxiety, depression, short sleep duration (average sleep duration less than 6 h), and poor sleep quality (a PSQI score of more than 5). We used logistic regression to evaluate the association between migraine and EDS according to headache frequency and calculated odds ratios (ORs) and their 95 % confidence intervals (CIs). We compared the clinical characteristics of participants with migraine with and without EDS using Student’s *t*-test for continuous variables and the chi-square test for categorical variables. *p*-values of <0.05 were considered statistically significant. The results were analysed using the statistical software SPSS 22.0 (IBM, Armonk, NY, USA).

As with most survey studies, missing data from a lack of responding occurred on several items. The data reported are the available data and therefore some variables deviate from the overall sample size of *n* = 2,695. Imputation techniques were not employed to minimize non-response effects [31].

## Results

### Survey

**Fig. 1:**
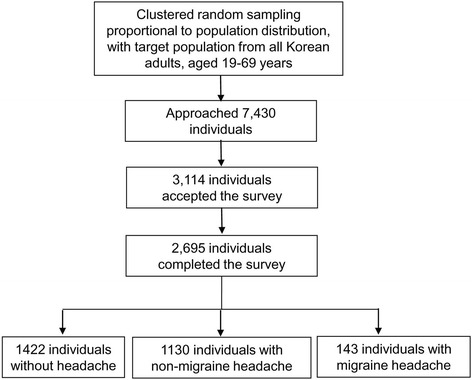
Flow chart depicting participation in the Korean Headache-Sleep Study

Our interviewers approached 7,430 individuals and 3,114 of them agreed to take the survey. Of them, 2,695 subjects completed the survey (Fig. 1). The distribution of age, gender, size of residential area, and educational level were not significantly different from those of the Korean general population (Table 1).

**Table 1:** Sociodemographic distribution of all survey participants including migraine, non-migraine headache, and excessive daytime sleepiness

	Sample number	Total population	*p*-value	Migraine	Non-migraine headache	Excessive daytime sleepiness
	N (%)	N (%)		N, % (95 % CI)	N, % (95 % CI)	N, % (95 % CI)
Gender						
Men	1345 (49.3^a^)	17,584,365 (50.6)	0.854^b^	36, 2.7 (1.8–3.5)	471, 35.0 (32.4–37.6)	161, 12.0 (10.2–13.7)
Women	1350 (50.7 ^a^)	17,198,350 (49.4)		107, 7.9 (6.5–9.4)	659, 49.0 (46.2–51.5)	152, 11.3 (9.6–12.9)
Age						
19–29	542 (20.5 ^a^)	7,717,947 (22.2)	0.917^b^	25, 4.5 (2.7–6.2)	231, 42.6 (38.4–46.8)	52, 9.6 (7.1–12.1)
30–39	604 (21.9 ^a^)	8,349,487 (24.0)		42, 7.0 (4.9–9.1)	269, 44.5 (40.6–48.5)	71, 11.8 (9.2–14.3)
40–49	611 (23.1 ^a^)	8,613,110 (24.8)		39, 6.5 (4.5–8.4)	277, 45.3 (41.4–49.3)	62, 10.1 (7.7–12.5)
50–59	529 (18.9 ^a^)	6,167,505 (17.7)		22, 4.1 (2.4-5.9)	204, 38.6 (34.4-42.7)	66, 12.5 (9.7-15.3)
60–69	409 (15.6 ^a^)	3,934,666 (11.3)		15, 3.9 (2.0–5.7)	149, 36.4 (31.7–41.1)	62, 15.2 (11.7–18.6)
Size of residential area						
Large city	1248 (46.3 ^a^)	16,776,771 (48.2)	0.921^b^	76, 6.1 (4.8–7.5)	525, 42.1 (39.3–44.8)	150, 12.0 (10.2–13.8)
Medium-to-small city	1186 (44.0 ^a^)	15,164,345 (43.6)		48, 4.0 (2.9–5.2)	488, 41.1 (38.3–43.9)	134, 11.3 (9.5–13.1)
Rural area	261 (9.7 ^a^)	2,841,599 (8.2)		19, 7.4 (4.2–10.6)	117, 44.8 (38.8–50.9)	29, 11.1 (7.3–14.9)
Educational level						
Middle school or less	393 (14.9 ^a^)	6,608,716 (19.0)	0.752^b^	22, 5.5 (4.2–7.7)	165, 42.0 (37.1–46.9)	58, 14.8 (11.2–18.3)
High school	1208 (44.5 ^a^)	15,234,829 (43.8)		50, 5.0 (3.8–6.3)	502, 41.6 (38.8–44.3)	135, 11.2 (9.4–13.0)
College or more	1068 (39.6 ^a^)	12,939,170 (37.2)		60, 5.6 (4.3–7.0)	457, 42.7 (39.8–45.8)	119, 11.1 (9.3–13.0)
Not responded				1, 3.8 (0.0–11.8)	6, 5.3 (0.3–9.6)	1, 3.8 (0.0–11.8)
Total	2695 (100.0 ^a^)	34,782,715 (100.0)		143, 5.3 (4.5–6.2)	1130, 41.9 (40.0–43.8)	313, 11.6 (10.4–12.8)

*CI* Confidence Interval

^a^Adjusted after age, gender, size of residential area and educational level

^b^Compared gender, age group, size of residential area, and educational level distributions between the sample of the present study and total population of Korea

### Prevalence of migraine and non-migraine headache

Of the 2,695 respondents, 143 (5.3 %) met the ICHD-2 criteria for migraine and 1,130 (41.9 %) were classified as having non-migraine headache. Finally, 1,422 participants (52.8 %) were classified as non-headache controls.

### Excessive daytime sleepiness and migraine

**Fig. 2:**
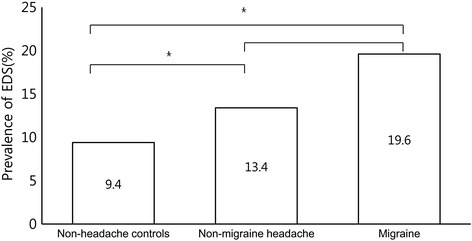
Comparison of the prevalence of EDS among participants *p < 0.05

Three hundred and thirteen (11.6 %) subjects reported ESS scores in an excess of 11 and were therefore classified as having EDS (Table 1). Among the 143 participants with migraine, 28 (19.6 %) were classified as also having EDS. Of the 1,130 participants with non-migraine headache and the 1,422 classified as non-headache controls, 151 (13.4 %) and 134 (9.4 %) were classified as having EDS, respectively. The prevalence of EDS was significantly higher among participants with migraine (OR = 2.3, 95 % CI = 1.5–3.7, *p* < 0.000) and non-migraine headache (OR = 1.5, 95 % CI = 1.2–1.9, *p* = 0.002) compared to non-headache controls. However, the prevalence of EDS was not significantly different between participants with migraine and those with non-migraine headache (OR = 1.6, 95 % CI = 1.0–2.5, *p* = 0.055) (Fig. 2).

Univariable logistic regression revealed that EDS prevalence among participants with migraine (OR = 2.3, 95 % CI = 1.5–3.7, *p* < 0.001) and non-migraine headache (OR = 1.5, 95 % CI = 1.2–1.9, *p* = 0.002) had increased ORs for EDS compared to non-headache controls. Anxiety (OR = 2.4, 95 % CI = 1.7–3.2, *p* < 0.001), depression (OR = 6.2, 95 % CI = 4.2–9.1, *p* < 0.001), short sleep duration (OR = 1.4, 95 % CI = 0.9–1.9, *p* = 0.122), and poor sleep quality (OR = 3.3, 95 % CI = 2.6–4.3) also had increased ORs for EDS. In the multivariable logistic regression for EDS adjusting for anxiety and depression (Model 1), migraine was not associated with increased ORs for EDS. After adjusting for short sleep duration (<6 h) and poor sleep quality (PSQI score > 5) (Model 2), however, non-migraine headache and migraine were revealed to have statistically significant ORs for EDS. The final model adjusting for anxiety, depression, short sleep duration, and poor sleep quality (Model 3), indicated that those with non-migraine headache and migraine did not have statistically significant ORs for EDS relative to non-headache controls (Table 2).

**Table 2:** Logistic regression to examine excessive daytime sleepiness by headache condition

	Univariable analysis for EDS		Multivariable analysis, adjusted for anxiety and depression (Model 1)		Multivariable analysis, adjusted for sleep duration and PSQI Score (Model 2)		Multivariable analysis, adjusted for anxiety, depression, sleep duration and PSQI score (Model 3)	
	OR	*P*-value	OR	*P*-value	OR	*P*-value	OR	*P*-value
Non-migraine headache	1.5 (1.2–1.9)	0.002	1.3 (1.0–1.7)	0.042	1.3 (1.0–1.7)	0.037	1.2 (0.9–1.5)	0.175
Migraine	2.3 (1.5–3.7)	<0.001	1.6 (1.0–2.6)	0.069	1.8 (1.2–2.9)	0.010	1.4 (0.8–2.2)	0.206
Anxiety	2.4 (1.7–3.2)	<0.001	1.3 (0.9–1.9)	0.135			1.2 (0.8–1.7)	0.504
Depression	6.2 (4.2–9.1)	<0.001	4.8 (3.1–7.4)	<0.001			3.7 (2.4–5.8)	<0.001
Short sleep duration (<6 h)	1.4 (0.9–1.9)	0.122			0.8 (0.5–1.2)	0.790	0.8 (0.5–1.1)	0.143
PSQI (PSQI score >5)	3.3 (2.6–4.3)	<0.001			3.3 (2.5–4.3)	<0.001	3.0 (2.2–3.8)	<0.001

*EDS* Excessive daytime sleepiness, *PSQ*I Pittsburgh Sleep Quality Index

### Excessive daytime sleepiness according to the headache frequency of migraine

Although not statistically significant, there was a tendency towards increased prevalence of EDS in migraineurs with at least 10 attacks per month (OR = 1.5, 95 % CI = 0.4–5.8, *p* = 0.557) compared to those with less than one attack a month. The ESS scores were not significantly different among migraineurs with less than one attack per month (6.2 ± 3.8), 1–9 attacks per month (7.4 ± 4.6), or at least 10 attacks per month (7.2 ± 4.9, *p* = 0.330).

### Clinical characteristics of subjects having migraine with and without EDS

We investigated the headache characteristics, headache frequency, VAS score for pain intensity, HIT-6 scores, psychiatric comorbidities such as anxiety and depression of participants with migraine grouped according the presence of EDS. Unilateral pain (39.3 % vs. 60.9 %, *p* = 0.039) was less prevalent and moderate-to-severe pain intensity (96.4 % vs. 76.5 %, p = 0.017) was more prevalent among migraineurs with EDS compared to migraineurs without EDS. Additionally, VAS scores for pain intensity (6.9 ± 1.8 vs. 6.0 ± 1.9, p = 0.014), HIT-6 scores (59.8 ± 10.2 vs. 52.5 ± 8.2, p < 0.001) and the prevalence of depression (42.9 % vs. 10.4 %, p < 0.001) was higher in migraineurs with EDS than those without EDS. The other measures did not significantly differ with the presence of EDS (Table 3).

**Table 3:** Clinical characteristics and impact of migraine according to excessive daytime sleepiness

	Migraine with EDS, *N* = 28	Migraine without EDS, *N* = 115	*P*-value
Headache characteristics			
Unilateral pain, N (%)	11 (39.3)	70 (60.9)	0.039
Pulsating quality, N (%)	18 (64.3)	90 (78.3)	0.123
Moderate-to-severe pain intensity, N (%)	27 (96.4)	88 (76.5)	0.017
Aggravation by movement, N (%)	23 (82.1)	77 (67.0)	0.116
Nausea, N (%)	23 (82.1)	102 (88.7)	0.349
Vomiting, N (%)	11 (39.3)	44 (38.3)	0.920
Photophobia, N (%)	16 (57.1)	68 (59.1)	0.848
Phonophobia, N (%)	23 (82.1)	78 (67.8)	0.136
Osmophobia, N (%)	14 (50.0)	54 (47.0)	0.722
Headache frequency, mean ± SD	5.1 ± 7.2	3.5 ± 5.9	0.178
VAS for pain intensity, mean ± SD	6.9 ± 1.8	6.0 ± 1.9	0.014
HIT-6 score, mean ± SD	59.8 ± 10.2	52.5 ± 8.2	<0.001
Anxiety, N (%)	11 (39.3)	32 (27.8)	0.236
Depression, N (%)	12 (42.9)	12 (10.4)	<0.001

*EDS* Excessive daytime sleepiness, *VAS* Visual Analogue Scale, *HIT-6* Headache Impact Test-6

## Discussion

The key findings in the present study were: (1) the prevalence of migraine and EDS in the Korean general population were 5.3 % and 11.6 %, respectively; (2) the prevalence of EDS was significantly higher among participants with migraine (19.6 %) and non-migraine headache (13.4 %) compared to non-headache controls (9.4 %); and (3) some clinical characteristics of migraine were more profound among migraineurs with EDS compared to migraineurs without EDS.

The 1-year prevalence of migraine in the present study was 5.3 % (2.7 % for men and 7.9 % for women). The 1-year prevalence of migraine in Asian countries has been found to range between 4.7 % and 9.1 % in most previous studies [32]. Our findings are similar to those reported in previous studies from Asian countries. The prevalence of migraine in Asia is relatively lower than that of western countries.

We found that the prevalence of EDS was 11.6 % in the Korean general population. Previous population-based studies have reported EDS using ESS mostly ranging from 10 % to 20 %. Three Australian studies among adults reported that EDS prevalence ranged from 11.7 % to 15.3 % [33–35]. A study in Singapore revealed an EDS prevalence of 9.0 % [36]. A previous study using data from the Korean Genome Epidemiology Study found that the prevalence of EDS was 12.2 % [37]. Our study showed a similar EDS prevalence to that from previous studies and therefore verified the reliability of the sampling and EDS diagnosis in our study.

Excessive daytime sleepiness has been reported to be associated with poor sleep quality and psychiatric conditions [38, 39]. Poor sleep quality is reported to be associated psychiatric conditions [40–42]. Furthermore, migraineurs were reported to have poor sleep quality and higher psychiatric comorbid conditions compared to non-headache controls [43–45]. In the present study, participants with migraine had an increased OR for EDS compared to non-headache controls in the univariable regression analysis. After adjusting for anxiety, depression, short sleep duration, and poor sleep quality, migraine was no longer a significant predictor of EDS while poor sleep quality and depression maintained significant associations with EDS (Table 3). Previous case-controlled studies have demonstrated similar findings. EM and CM had increased ORs for EDS in a univariate analysis [15, 20]. However, after adjusting for poor sleep quality, drugs, and gender, EM and CM were no longer statistically significant. These findings suggest that a significant association between migraine and EDS in a univariable analysis may be attributable to poor sleep quality and/or depression among migraineurs rather than the migraine itself.

In the present study, migraineurs with EDS experienced more severe headache intensity, reported a higher impact of the headache, and more depressive symptomology than those without EDS. These findings suggest that migraineurs with EDS have more severe clinical burden than migraineurs without EDS. Considering findings in the present study, physicians should carefully investigate the level of EDS and factors that exacerbate it, such as poor sleep quality and depressive symptomology when caring for patients with migraine.

This study had some limitations. First, although our research is a large population-based study with low sampling error, its statistical power for executing a subgroup analysis was limited. Some results might not have reached statistical significance because of the limited sample size. Second, this study is cross-sectional and the directionality of the association is uncertain. It cannot be determined whether migraine causes EDS or vice versa. Third, we surveyed the sleep habits of participants based solely on self-report. Further, we did not use any tool to evaluate characteristics of sleep such as polysomnography, which could further explore the sleep architecture, total sleep time, sleep efficiency, or other relevant sleep variables among participants with EDS.

Our study has several strengths. First, this study has a large sample size in a population-based setting and the estimated sampling error was low. Second, we investigated risk factors for EDS after adjusting for sleep duration. Third, we assessed the clinical features of migraine in participants with EDS compared with those without EDS.

## Conclusions

In summary, migraineurs had a higher prevalence of EDS relative to non-headache controls. Further, migraineurs with EDS experienced a more severe headache, depressive mood, and higher impact of headache. The presence of EDS should be carefully evaluated in migraineurs, in order to relieve the headache as well as reduce the impact of headache.

## Abbreviations

EDS, excessive daytime sleepiness; ESS, epworth Sleepiness Scale; VAS visual Analogue Scale; OSA, obstructive sleep apnea; EM, episodic migraine; CI, confidence interval; CM, chronic migraine; ICHD, International Classification of Headache Disorders; KHSS, Korean Headache-Sleep Study; PSQI, Pittsburgh Sleep Quality Index score; GAS, Goldberg Anxiety Scale; PHQ-9 Patient Health Questionnaire-9; HIT-6 Headache Impact Test-6; OR odds ratio
